# Quality Status Analysis and Intrinsic Connection Research of Growing place, Morphological Characteristics, and Quality of Chinese Medicine: Cyperi Rhizoma (Xiangfu) as a Case Study

**DOI:** 10.1155/2022/8309832

**Published:** 2022-03-21

**Authors:** Junrong Lu, Wenbing Li, Na Xu, Peifen Yao, Shengpeng Wang, Chaomei Fu, Jin Pei, Hulan Chen, Shu Wang

**Affiliations:** ^1^West China School of Pharmacy, Sichuan University, Chengdu 610041, Sichuan, China; ^2^College of Pharmacy, Chengdu University of Traditional Chinese Medicine, Chengdu 611137, Sichuan, China; ^3^Sichuan Provincial Qiang-Yi Medicinal Resources Protection and Utilization Technology Engineering Laboratory, Institute of Qinghai-Tibetan Plateau, Southwest Minzu University, Chengdu 610225, Sichuan, China; ^4^State Key Laboratory of Quality Research in Chinese Medicine, Institute of Chinese Medical Sciences, University of Macau, Macao, China

## Abstract

**Materials and Methods:**

The macroscopic characteristics of CR as well as its moisture, ethanol extract, essential oil, total ash, and acid-insoluble ash contents were examined and calculated. In addition, qualitative identification and quantitative determination of *α*-cyperone, cyperotundone, and nootkatone were simultaneously performed, and a high-performance liquid chromatography (HPLC) fingerprint chromatogram was established. Inductively coupled plasma mass spectrometry and gas chromatography methods recorded in ChP were used to measure the contents of residues of heavy metal and deleterious elements as well as residues of organochlorine pesticide, respectively. Hierarchical cluster analysis and typical canonical correlation analysis were performed using Origin 9.1 and SPSS 23.0 to explore the correlation between CR's growth area, morphological characteristics, and quality.

**Results:**

Of the 47 batches of CR analyzed, only 4 collected from the province of Shandong had a flat appearance, which did not accord with the macroscopic characteristics of CR. Overall, only 4 batches met ChP standards for CR. In addition, 30 and 38 batches did not meet the requirements for moisture content and essential oil content, respectively. The similarity values of HPLC fingerprints ranged from 0.568 to 0.986. Results of hierarchical cluster analysis for ethanol extracts, essential oil, *α*-cyperone, cyperotundone, and nootkatone and the HPLC fingerprints (total peak time and peak area) suggested that the samples could be classified into four clusters, with no significant difference in growth geographic areas among them. Results of canonical correlation analysis indicated that the first canonical pair could represent the correlation between macroscopic characteristics (vector 1) and chemical quality (vector 2), with shorter diameter and length denoting lower ethanol extract content and higher nootkatone content in a single grain of CR.

**Conclusions:**

Crude medicinal materials were collected and examined in this study to reflect the overall quality status of CR in China. The methods chosen to detect, calculate, and analyze the quality of CR were suitable to the investigation, and the results are crucial not only for estimating the current quality status of CR, but also for conducting further research into its cultivation, quality assurance, and commodity specification. Besides, this mode of investigation could be used to evaluate other medicines.

## 1. Introduction

### 1.1. Basic Information and Application of CR

The efficacy of an herbal medicine depends on its quality, which is influenced by such factors as place of origin, climate, time, and manner by which the herb is cultivated, collected, processed, and preserved. Cyperi Rhizoma (CR) is a commonly used medicine in clinic that is well recognized and extensively used worldwide. It is the dried rhizome or tuber of *Cyperus rotundus* L. of Cyperaceae, a grass-like erect perennial herb that can grow up to 95–150 cm in height [1]. Its application, usage, and dosage vary per healthcare system in different countries or regions therein [2–4]. For instance, CR has been used as a herbal remedy to treat bowel and stomach disorders in India, Thailand, Iran, Sudan, and Japan for centuries [5–8].

In traditional Chinese medicine (TCM), it was first recorded as *Suocao gen* in the classic herbal reference “*Ming Yi Bie Lu*.” At present, it is referred to as *Xiangfu* or *Xiangfu zi*. It grows worldwide on hillsides in uncultivated grass or in damp places near water. In China, it is produced in many provinces, including Shanxi, Gansu, Henan, Shandong, Jiangxi, Anhui, Sichuan, Guangdong, and Guangxi, which belong to different geographic areas with significant climatic differences [1]. According to TCM theory, CR is pungent, slightly bitter and slightly sweet in flavor, and neutral in nature and acts on the liver, spleen, and San Jiao channels. It soothes the liver to resolve depression, moves Qi to soothe the middle Jiao, regulates menses, and relieves pain [9]. The “*Ben Cao Bei Yao*” collection recorded it as a general medication for treating Qi disease and the primary medication for treating gynecological disease (*Qi Bing Zhi Zong Si*, *Nv Ke Zhi Zhu Shuai*). It has long been included in such prescriptions as *Cai Hu Su Gan San* and *Yue Ju Wan*, which are very popular formulas for treating diseases caused by Qi dysfunction. In addition, 107 preparations containing CR are listed in the 2015 edition of the Chinese Pharmacopeia (ChP), indicating that 7.14% of prepared preparations and single medicinal preparations are made of CR combined with other Chinese herbal medicines.

### 1.2. Chemical Components and Pharmacological Activities of CR

The major chemical constituents of CR include essential oils, total phenol, flavonoids, terpenoids, sesquiterpenes, phenylpropanoids, phenolic acids, alkaloids, iridoid glycosides, and saponins [10]. Previous pharmacological studies on CR have reported that methanol (MeOH) extract, ethanol extract, aqueous extract, and some components isolated from CR have exhibited pharmacological functions, such as analgesic, free radical scavenging, antiandrogenic, antimicrobial, cytotoxic, anticonvulsant, antidiabetic, antigenotoxic, anti-inflammatory, antiplatelet, antiuropathogenic, hepatoprotective, lactogenic, antidepressant, neuroprotective, nootropic, antilipidemic, and cardioprotective activities [6, 11–19]. Total MeOH extract, from which three components could be isolated, namely, methyl 3,4-dihydroxybenzoate, ipolamiide, and 6b-hydroxyipolamiide, was reported to have hepatoprotective activity against CCl_4_-induced hepatic injury [20]. The 70% ethanol extract of CR was found to have the most potent antioxidant activity for metabolites involved in it, such as polyphenols, flavonoids, and sesquiterpenes [10]. The aqueous extract of CR could stimulate the synthesis of prolactin significantly, which could increase milk secretion [21]. *α*-Cyperone could inhibit LPS-induced inflammation in RAW 264.7 cells by regulating cyclooxygenase-2 expression, prostaglandin *E*_2_ production, and nuclear factor *κ*B signaling. Research has also shown that nootkatone has a potent inhibitory effect on collagen-, thrombin-, and arachidonic acid–induced platelet aggregation and prolonged mice tail bleeding times [13].

### 1.3. Significance of CR

The original plant of CR is regarded as a stubborn and obnoxious weed widely distributed in vegetable grounds for which different herbicides and various methods have been employed [22–26]. Because of the high dosage and unscientific use of herbicides, large-scale production of CR's natural raw herb is now rarely seen, even in some traditional production areas. According to our field investigation, a sustainable production volume of CR could be hardly maintained even in main production areas, such as the provinces of Guangdong, Guangxi, and Hainan, because of large price fluctuations and the resulting relatively low prices and profits. These circumstances affect not only the quantity status of CR, but also its quality and clinical efficacy. Furthermore, although it is a well-known traditional Chinese herbal medicine, it is not listed in mainstream herbal pharmacopoeia standards, such as the United States Pharmacopoeia, British Pharmacopoeia, European Pharmacopoeia, and Japanese Pharmacopoeia. It is currently only recorded in ChP, albeit with very apparent shortages. For example, the only indicator for content determination cited is the volume of essential oil, without any specific chemical components. In addition, the contents of heavy metal and deleterious elements as well as the residues of organochlorine pesticide, which are key factors in determining the classification and exporting of crude Chinese herbal medicines, are not included. Moreover, the correlation between area of origin and quality of CR is hardly reported in the literature, let alone the correlation between its macroscopic characteristics and chemical quality.

In a previous study, we used thin-layer chromatography (TLC) and high-performance liquid chromatography (HPLC) to qualitatively and quantitatively determine indicator components of CR, namely, cyperotundone, *α*-cyperone, and nootkatone [27]. In this study, we further analyzed the quality status of CR comprehensively based on 47 batches of CR from 12 provinces across six geographic divisions in China and investigated several key factors that highly related to the quality of CR. An HPLC fingerprint chromatogram was established to estimate the total chemical components of CR and their differentiation. In addition, hierarchical cluster analysis (HCA) and typical canonical correlation analysis (CCA) were used to analyze the correlation between the habitat of CR and its quality and that between its macroscopic characteristics and chemical quality, respectively. Our comprehensive evaluation provides cause for further research development and a prospective approach for studying other traditional Chinese herbal medicines.

## 2. Materials and Methods

### 2.1. Chemicals, Reagents, and Crude Medicinal Materials

The reference standards of cyperotundone, *α*-cyperone, and nootkatone (purity, ≥98%) were purchased from Chengdu Pufei De Biotech (Chengdu, China). HPLC grade MeOH was purchased from Themo Fisher Scientific (Waltham, USA). Distilled water was obtained from Watersons (Guangzhou, China). Standard solution of Pb, Cd, Hg, As, and Cu was obtained from national research center for reference materials (Beijing, China) with the mass concentration 1000 *μ*g·mL^−1^. Other reagents were of analytical purity grade. Forty-seven batches of CR were personally gathered by our laboratory staff or entrusted to others from the 12 provinces explored, including Shandong, Sichuan, Hainan, Guangdong, Jiangxi, Anhui, and Yunnan. The samples were authenticated by Professor JinPei (Chengdu University of Traditional Chinese Medicine, Sichuan, Chengdu, China). All samples were stored at the Chengdu University of Traditional Chinese Medicine Pharmacy School.

### 2.2. Quality Indexes Assessed according to ChP

The macroscopic characteristics, such as shape, color, length, diameter, and weight, of the samples were recorded by observing and measuring 90 single grains from every batch of CR. The contents of moisture, total ash, ethanol extracts, and essential oil were determined according to indexes 0832 (fourth method), 2302, 2201, and 2204, respectively, of Volume IV of ChP (2015 Edition), which considers these indexes as national standards of CR.

The TLC identification method previously established by our team was used to simultaneously examine cyperotundone, *α*-cyperone, and nootkatone in every batch of CR [27]. In brief, dried and powdered CR (1 g) was ultrasonically extracted (250 W, 40 kHz) with 20 mL of MeOH. The MeOH solution was filtered, and the filtrate was allowed to dry. The residue was dissolved in MeOH (1 mL) as the sample solution. Cyperotundone, *α*-cyperone, and nootkatone were dissolved in MeOH at a concentration of 1 mg·mL^−1^ as the reference solution. Two microliters of each solution was absorbed on the same silica gel GF254 laminate. Petroleum (60–90°C) and ethyl acetate were chosen as developing agents at a ratio of 9: 1 (vol/vol). After being developed, the laminates were taken out, dried, and examined under ultraviolet light at 254 nm. Before being examined under ultraviolet light at 365 nm, the laminates were sprayed with 10% sulfuric acid in ethanol for color development and heated at 105°C until color is shown.

The contents of acid-insoluble ash, residues of heavy metal and deleterious elements, and residues of organochlorine pesticide were detected according to indexes 2302, 0412 (inductively coupled plasma mass spectrometry), and 2341 (second method for pesticide residues; gas chromatography), respectively, of Volume IV of ChP 2015 Edition.

### 2.3. Determination of Specific Chemical Components and Establishment of Fingerprint Chromatogram of CR by HPLC

The preparation method for the samples, standard solution, and chromatographic conditions used to detect the three specific chemical components of CR, namely, cyperotundone, *α*-cyperone, and nootkatone, was performed according to our previous research [27]. Briefly, dried and powdered CR was precisely weighed (0.5 g) and immersed in 25 mL of MeOH. Additional MeOH was added to make up for the loss thereof after ultrasonic extraction (250 W, 40 kHz) for 30 min. The filtrate was filtered (0.45 *μ*m pore size) prior to injection for HPLC analysis. The standard substances of *α*-cyperone, cyperotundone, and nootkatone were precisely weighed and dissolved in chromatographic MeOH. HPLC determination was performed on a Phenomenex C_18_ chromatographic column (4.6 mm × 250 mm, 5 *μ*m). The mobile phase consisted of MeOH and water at a ratio of 68:32 (vol/vol). The flow rate was 1.0 mL·min^−1^, and the injection volume was 10 *μ*L. The column temperature was set at 30°C, and the wavelength was monitored at 242 nm.

The HPLC method utilized to establish a fingerprint chromatogram of CR was performed as described in the literature [28]. In brief, powdered CR was precisely weighed (0.5 g) and mixed in 10 mL of MeOH. Additional MeOH was added to make up for the loss thereof after ultrasonic extraction (160 W, 59 kHz) for 30 min. The filtrate was filtered (0.45 *μ*m pore size) prior to injection for HPLC analysis. The standard substances of *α*-cyperone, cyperotundone, and nootkatone were precisely weighed and dissolved in chromatographic MeOH. HPLC determination was performed using a Phenomenex C_18_ chromatographic column (4.6 mm × 250 mm, 5 *μ*m). The mobile phase consisted of MeOH (A) and water (B) with the gradient elution for 0–45 min (40% to 80% A) and 45–60 min (80% A). The flow rate was 1.0 mL·min^−1^, and the injection volume was 10 *μ*L. The column temperature was set at 25°C, and the wavelength was monitored at 254 nm.

### 2.4. Data Analysis

Data obtained from experiments were analyzed using Origin 9.1 (Northampton, USA) and IBM SPSS 23.0 (Armonk, USA) for HCA and CCA, respectively.

Graphs in this paper like radar graph were drawn by Origin 9.1 (Northampton, USA) to show the contents of moisture, total ash, essential oil, residues of heavy metal and deleterious elements, and residues of organochlorine pesticide, etc.

## 3. Results

### 3.1. Macroscopic Characteristics of CR

**Figure 1:**
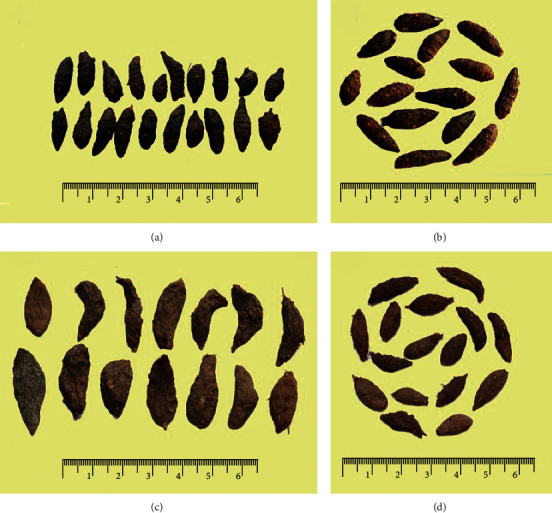
Appearance shape of CR. Some batches of CR are chosen to express the difference on appearance shape. (a, b) Usual shape of S44, S30 batch of CR with spindle shape, which is recorded in ChP. (c, d) Unusual shape of S34 and S35 batch of CR with deplanate shape.

Most batches of CR met ChP standards, with spindle shape (Figures 1(a) and 1(b)) length ranging from approximately 2 to 3.5 cm and diameter from approximately 0.5 to 1 cm, as well as other stipulations on surface color, cross-section color, and their features, such as odor and taste. Four batches (S09, S10, S34, and S35) obtained from Shandong had a clearly flat shape (Figures 1(c) and 1(d)). The mean length and diameter of CR were 2.1 and 0.6 cm, respectively. Some batches of CR collected from Jiangxi (S02 and S03), Guangdong (S04, S05, S06, and S31), and Guangxi (S12, S22, and S25) had a length shorter than 2 cm. The average weight of a single grain was 0.5567 g. One-way Analysis of Variance (ANOVA) method in SPSS software was used to analyze the differences in the length, diameter, and weight of 90 single grains of CR of every batch. The result showed that, compared with Batch S01, approximately 34, 26, and 38 of the 47 batches of CR showed significant differences in length, diameter, and weight, respectively. Places of origin and macroscopic characteristic indexes are detailed in Table 1.

### 3.2. Examination, Extracts, and Content Determination Indexes of CR in ChP

**Figure 2:**
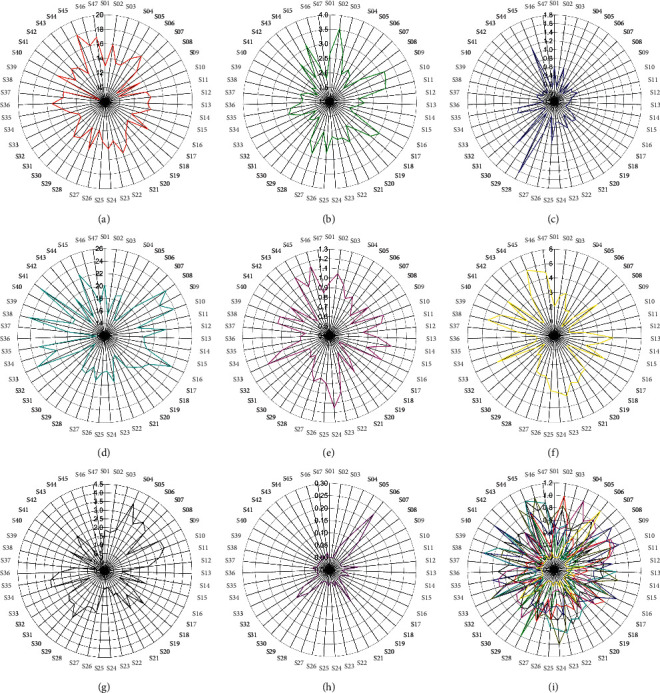
The radar graph of contents detected in CR: (a) moisture content, (b) total ash content, (c) acid-insoluble ash content, (d) ethanol extracts content, (e) essential content, (f) cyperotundone content, (g) *α*-cyperone content, (h) nootkatone content. A to H show the contents of every single batch of CR originally and separately. (i) All the indexes after being standardized processed and presented together. All lines of these radar graphs are jagged shape, which indicate the big differences among 47 batches of CR.

According to Volume I of ChP (2015 Edition), all CR samples met the standard for total ash content of less than 4.0%, but only 17 batches met that for moisture content of less than 13.0%. On the ethanol extract contents of all batches, only S05, S31, S36, and S42 were lower than 15.0% and hence did not meet the standard. In addition, the average of essential oil contents of all batches was 0.83%, ranging from 0.52% to 1.15%, with only 9 batches meeting the standard for essential oil of approximately more than 1.0% (in milliliters per Gram). Overall, only 4 of the 47 batches fully met the standards of CR in ChP (2015 edition), namely, S10, S16, S33, and S44, which originated from Shandong, Shanxi, Guangdong, and Guangxi, respectively. The moisture, total ash, ethanol extract, and essential oil contents are shown in Figures 2(a), 2(b), 2(d), and 2(e).

### 3.3. TLC Identification

**Figure 3:**
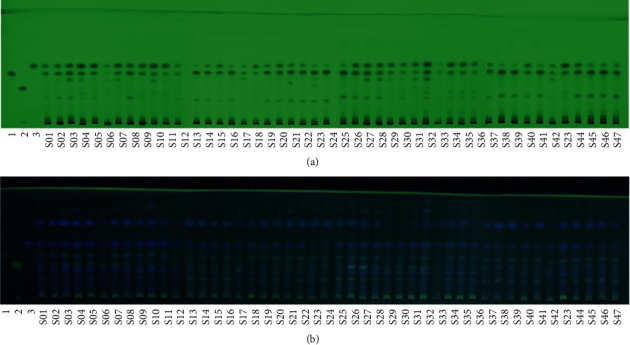
The TLC fingerprint of CR samples no. S01–S47 under ultraviolet light 254 nm (a) and 365 nm (b). Reference solution of cyperotundone (1), nootkatone (2), and *α*-cyperone (3). S01–S47 show CR samples from S01 to S47, respectively.

Results showed that the spots of reference standards could be observed clearly under ultraviolet light at 254 and 365 nm. In CR samples, cyperotundone and *α*-cyperone could be well separated and visible under 254 and 365 nm (Figure 3). Comparing the depth and size of spots in 47 batches of CR samples, it is obvious that the content of cyperotundone and *α*-cyperone in some batches was relatively low. However, the spots of nootkatone in 47 batches of CR samples are visible only under ultraviolet light at 365 nm (Figure 3(b)), not visible at 254 nm (Figure 3(a)), suggesting that the nootkatone content in these batches was very low. These detected compounds were structural isomers. The results indicate that their contents accumulated differently under various growth conditions, which highlights the importance of studying the correlation between the quality and growth environment of CR.

### 3.4. Contents of Acid-Insoluble Ash, Residues of Heavy Metal and Deleterious Elements, and Residues of Organochlorine Pesticide

**Figure 4:**
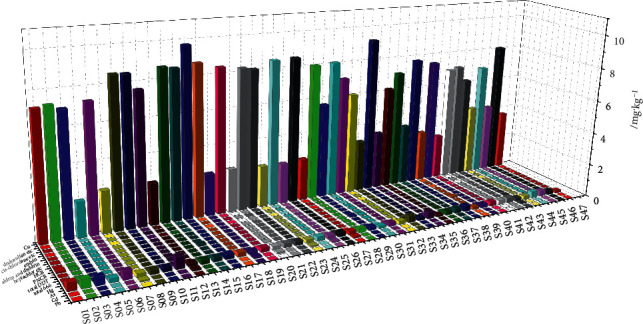
Results of residues of heavy metal and deleterious elements, and organochlorine pesticide (*n* = 3). Endosulfan etc. means the total content of endosulfan, endosulfan, and endosulfan sulfate. Cis-chlordane means the total content of cis-chlordane, trans-chloride, and oxy-chlordane. Heptachlor means the total of heptachlor, heptachlor-exo-epoxide, and heptachlor-endo-epoxide. HCB means the content of hexachlorobenzene. PNCB means the content of pentachloronitrobenzene. Total DDT means the total content of dichlorodiphenyltrichloroethane, namely, p,p′-dichlorodiphenyldichloroethylene, p,p′-dichlorodiphenyldichloroethane, o,p′-DDT, and p,p′-DDT. Total 666 means the total content of hexachlorocyclohexane (BHC), namely, *α*-BHC, *β*-BHC, *γ*-BHC, and *δ*-BHC. Ag, Cd, As, Hg, and Cu mean the contents of lead, cadmium, arsenic, Mercury, and copper, respectively. The tags of S01, S02, S03, etc. mean the samples of CR with the batch number of S01 to S47.

Acid-insoluble ash content was detected in the range of 0.03%–0.76% (Figure 2(c)), with a mean of 0.33%. With regard to the quality standards of other crude medicinal materials, such as Glycyrrhizae radix et rhizoma (*Gancao*), Lonicerae japonicae flos (*Jinyin hua*), and Astrgali radix (*Huangqi*), the contents of residues of heavy metal and deleterious elements and those of organochlorine pesticide in all samples met the standards outlined in ChP. The contents of lead, cadmium, arsenic, Mercury, and copper were lower than 5.0, 0.3, 2.0, 0.2, and 20.0 mg·kg^−1^, respectively. The total content of hexachlorocyclohexane (BHC; *α*-BHC, *β*-BHC, *γ*-BHC, and *δ*-BHC) was lower than 0.2 mg kg^−1^. The total content of dichlorodiphenyltrichloroethane (DDT; *p*,*p*′-dichlorodiphenyldichloroethylene, *p*,*p*′-dichlorodiphenyldichloroethane, *o*,*p*′-DDT, and *p*,*p*′-DDT) was lower than 0.2 mg·kg^−1^. The contents of quintozene (pentachloronitrobenzene) and hexachlorobenzene were lower than 0.1 mg·kg^−1^. The total contents of heptachlor, heptachlor-exo-epoxide, and heptachlor-endo-epoxide were lower than 0.05 mg·kg^−1^. The total contents of aldrin and dieldrin were lower than 0.05 mg·kg^−1^. The content of endrin was lower than 0.05 mg·kg^−1^. The total contents of *cis*-Chlordane, *trans*-Chloride, and oxychlordane as well as those of *α*-endosulfan, endosulfan, and endosulfan sulfate did not exceed 3.0 mg·kg^−1^. These results are illustrated in Figure 4.

### 3.5. Contents of *α*-Cyperone, Cyperotundone, and Nootkatone

**Figure 5:**
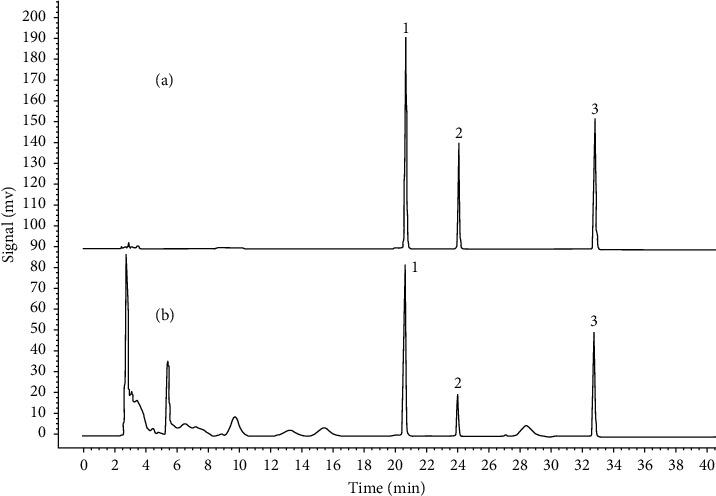
HPLC chromatogram of CR samples: (a) the mixed standard solution and (b) the sample solution of CR. Three specific components are cyperotundone (1), nootkatone (2), and *α*-cyperone (3), respectively.

Results demonstrated that the content of *α*-cyperone, cyperotundone, and nootkatone was significantly different in the 47 batches of CR, with the overall trend being cyperotundone > *α*-cyperone > nootkatone. The content ranged from 0.8037 to 4.2485 mg·g^−1^ for cyperotundone, from 0.4221 to 3.7346 mg·g^−1^ for *α*-cyperone, and from 0.0000 to 0.2370 mg·g^−1^ for nootkatone, with mean values of 2.5780, 1.7719, and 0.0338 mg g^−1^, respectively. Nootkatone could only be examined in 12 batches of samples. The contents are shown in Figures 2(f)–2(h). The HPLC chromatogram is shown in Figure 5.

### 3.6. HPLC Fingerprint Chromatogram

**Figure 6:**
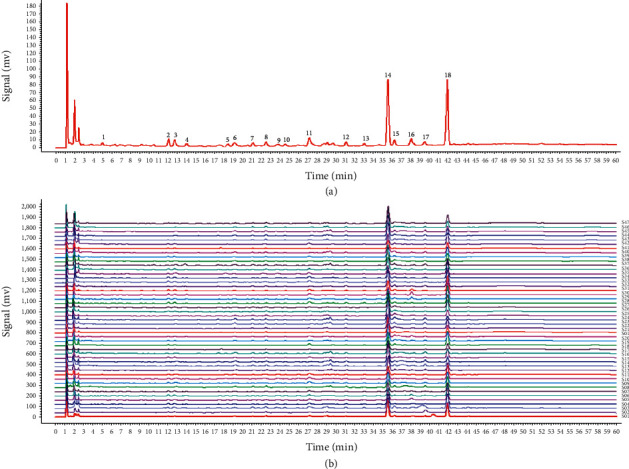
HPLC fingerprints of CR samples: (a) the total pattern of fingerprints and (b) the superimposed fingerprints of 47 batches of CR. The peak numbers of cyperotundone, *α*-cyperone, and nootkatone are pick 14, 18, and 16, respectively.

The 2012 version of the Similarity Evaluation System for Chromatographic Fingerprint of Chinese Materia Medica software was used for similarity analysis and characteristic peak analysis of the 47 batches of CR samples. The peak of cyperotundone was chosen as reference peak. Eighteen common characteristic peaks were screened by automatic matching. Three components were identified by the reference substance: cyperotundone (peak 14), *α*-cyperone (peak 18), and nootkatone (peak 16) (Figure 6). The similarity values of the samples ranged from approximately 0.568 to 0.986, indicating that CR circulated in markets with highly uneven quality.

### 3.7. HCA of CR Samples

Data on the 47 batches of CR samples were loaded into Origin 9.1 for cluster analysis to explore the correlation between quality and growth area. The cluster method and distance type were set as farthest neighbor and Euclidean, respectively. In addition, 25 batches collected from main production areas (Guangdong, Guangxi, and Hainan in the South China District) were also further processed using HCA.

#### 3.7.1. Content Analysis of Ethanol Extracts, Essential Oil, Cyperotundone, *α*-Cyperone, and Nootkatone

**Figure 7:**
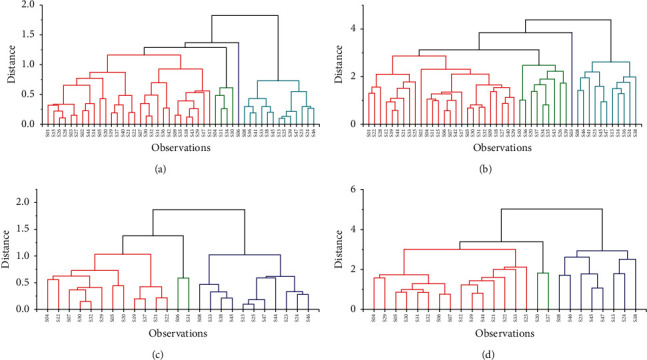
HCA results of CR samples. (a) HCA results of 47 batches of CR using data of the batch number, the contents of ethanol extracts, essential oil, cyperotundone, *α*-cyperone, and nootkatone. (b) HCA results of 47 batches of CR using data of the batch number, peak time, and peak area. (c) HCA results of 25 batches of CR growing in South China using data of the batch number, the contents of ethanol extracts, essential oil, cyperotundone, *α*-cyperone, and nootkatone. (d) HCA results of 25 batches of CR collected in South China using data of the batch number, peak time, and peak area.

The batch number as well as the content of ethanol extract, essential oil, cyperotundone, *α*-cyperone, and nootkatone were used for cluster analysis. When the Euclidean distance was more than 1.25, all 47 batches of CR could be divided into four categories (Figure 7(a)) and 25 batches from South China could be clustered into three (Figure 7(c)).

#### 3.7.2. Analysis of HPLC Fingerprints

The batch number, peak time, and peak area of CR were used for cluster analysis. More than 16 batches of CR for which the peak area could not be calculated were ruled out in HCA. When the Euclidean distance was more than 3, all 47 batches could be divided into four categories (Figure 7(b)), and 25 batches from South China could be clustered into three (Figure 7(d)).

Figure 7(a): Specific batch of CR in HCA results of 47 batches of CR using data of the batch number, the contents of ethanol extracts, essential oil, cyperotundone, *α*-cyperone, and nootkatone. Figure 7(b): Specific batch of CR in HCA results of 47 batches of CR using data of the batch number, peak time and peak area. Figure 7(c): Specific batch of CR in HCA results of 25 batches of CR growing in South China using data of the batch number, the contents of ethanol extracts, essential oil, cyperotundone, *α*-cyperone, and nootkatone. Figure 7(d): Specific batch of CR in HCA results of 25 batches of CR collected in South China using data of the batch number, peak time, and peak area.

The specific batch of CR in different categories was shown in Table 2. The HCA results processed on the above-cited five components and on HPLC fingerprints could not distinguish the 47 samples into different geographic areas. For example, category 2 in Figure 7(a) included batch of S04, S10, S11, and S34, which belong to three different geographical districts, South China, East China, and Central China. Further analysis of the HCA results for the samples from South China revealed that those from Guangdong, Guangxi, and Hainan could not be distinguished also. Overall, the quality of CR growing in different areas of China had no clear geographic distinction, which is consistent with the growth pattern of CR.

### 3.8. CCA of CR Samples

Data on the macroscopic characteristics (vector 1), length, diameter, weight, and indicators of chemical quality (vector 2), the contents of ethanol extracts, essential oil, cyperotundone, *α*-cyperone, and nootkatone of the 47 batches of CR were processed by CCA to determine the correlation between macroscopic characteristics and quality of CR.

The correlation coefficient between the first pair of canonical variables was 0.774, and Wilk analysis showed that only the first canonical variable was statistically significant (*P* < 0.001). The first typical variable of chemical quality (vector 2) could be understood as “length and size factor” as it was more primarily related to length and diameter (Table 3). The first typical variable of macroscopic characteristics (vector 1) was more primarily related to the content of ethanol extracts and nootkatone (Table 4). Through cross-load analysis of vectors 1 and 2, the first typical variable of chemical quality highly correlated with diameter and length, with shorter diameter and length indicating lower ethanol extract content and higher nootkatone content.

**Figure 8:**
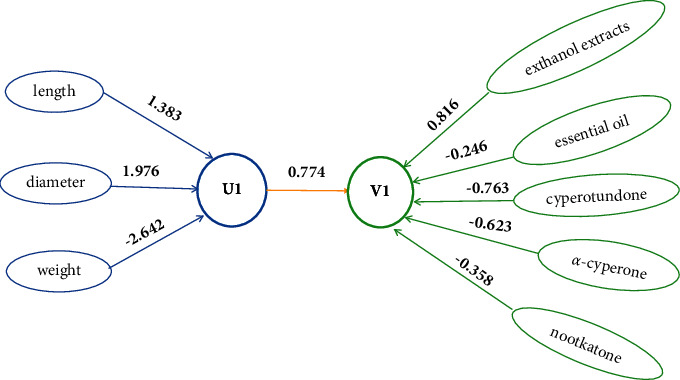
The typical structure diagram of CCA result. U1 means the first pair of canonical variables of macroscopics characters (gather one), and V1 means the first pair of canonical variables of chemical quality (gather two).

Through typical redundancy analysis, the first typical variable could explain 20.0% and 29.6% of the variance in vectors 1 and 2, respectively. Three pairs of canonical variables could explain 100% and 67.3% of the variance of the factors within vectors 1 and 2, respectively. The typical structure between vectors 1 and 2 is shown in Figure 8.

## 4. Discussion

The quality of traditional Chinese medicinal materials is the most important factor for their efficiency. In this study, we obtained 47 batches of CR samples from current main production areas (e.g., Guangdong and Guangxi), traditional high-quality production areas (e.g., Shandong), and other provinces where CR grows (e.g., Sichuan and Jiangxi) to represent the general condition of CR in the circulation market. Studying a large batch of CR comprehensively allowed us to evaluate its quality more accurately.

The curves in our radar diagrams are not smooth but rugged and sharp even when the standardized one is processed (Figure2(i)). Significant differences in the contents of moisture, total ash, acid-insoluble ash, ethanol extracts, essential oil, *α*-cyperone, cyperotundone, and nootkatone were detected per batch of samples. In light of the substantially high number of CR analyzed in this study, the quality of CR obtained from the market was not as good and stable as that of the samples we previously reported [29]. The low similarities of HPLC fingerprints further verified this disparity in quality. As the content of residues of heavy metal and deleterious elements, and residues of organochlorine pesticide of 47 batches of CR could meet the standard requirements outlined in ChP 2015 edition, high export potential of CR should be developed. In addition, much attention should be given to ensure the quality of CR by determining essential oil content and controlling moisture content. While 63.83% of the batches of samples exhibited higher than 13.0% moisture content, most batches (80.85%) of samples did not meet the standards for essential oil content of more than 1.0% (in milliliters per gram), implying that the low content of essential oil might not be due to high temperature or the length of time it took to dry the medicinal material. Thus, the maximum standard for essential oil content should be further investigated in an even higher number of batches to obtain improved results. *α*-Cyperone, cyperotundone, and nootkatone have been reported as the main components of CR with significant pharmacological activities [13, 14, 19, 20, 30]. Therefore, their specific and effective content in CR should be included in the national standards to assess CR's quality more accurately.

Genuine medicinal materials in TCM are regarded as having the best macroscopic characteristics and highest quality. Multicomponents, multitargets, and multilevels are typical features of traditional Chinese medicinal materials. Their quality could not be reflected by just one or several chemical components and is often related to their production area, which is why we used HCA to evaluate the correlation between them. However, given the limited availability of medicinal materials, the number of CR samples acquired from main production areas in South China, where highly automatic sowing, harvesting, and processing technologies are now being applied, was higher than that obtained from the other geographic divisions we explored. No significant difference in quality was found between the six geographic districts based on the HCA results for all 47 batches of CR samples and those for the 25 batches from South China, which echoes the wide distribution of the original plants of CR in the whole country. This in turn indicates that the upgrade to highly automatic technologies in main production areas of CR might be scientific and appropriate. However, consistency in the quality of CR needs to be better guaranteed by establishing standard production procedures (e.g., quality of seeds or rhizoma, sowing time, growing years, harvesting time, processing method, and parameters) in the future. What is more, the pharmacological study on efficacy differentiations of CR from different growth places also needs to be conducted.

Macroscopic characteristics often correlate with the chemical characteristics of crude medicinal materials. Regarding commercial grades of Chinese medicinal materials (T/CACM 1021.109-2018) established by the China Association of Chinese Medicine, CR is initially classified as “Mao CR” or “Guang CR.” “Guang CR” is then further classified into “picked” or “unpicked.” CR that falls under the “picked” category is finally classified into three grades based on the sieve it could pass. Histochemical analysis confirmed that the accumulation of primary and secondary metabolites varies in macro-dissected parts of herbal medicines [31–33]. Research has shown that ginseng “flesh” (xylem, phloem, and resin canal) has more polysaccharides with higher molecular weights and higher ratios of glucose residue, whereas ginseng “skin” (cork and cortex) has fewer polysaccharides with lower molecular weights and higher ratios of nonglucose constituents (e.g., galacturonic acid, galactose, arabinose, and rhamnose) [34]. Our CCA results verified that a lower ethanol extract content and a higher nootkatone content in CR are correlated with shorter diameter and length. The contents of ethanol extracts and nootkatone represent the general chemical composition and specific components of CR that can be extracted by their corresponding solvents. However, studies have yet to determine if there is any correlation between the morphological parameters of CR and its quality over time. In addition, the grade criteria of CR are only based on size, warranting immediate and further research into the correlation between its size and quality to ascertain the correlation between accumulation of primary and secondary metabolites and size as well as to establish a more appropriate grade standard closely related to quality. Histochemical analysis combined with other modern techniques would prove to be crucial in this regard.

## 5. Conclusions

In this study, we used a series of quality control methods that were suitable for comprehensive investigation of the quality of CR. The results could be used to estimate the current quality status of CR in China and ultimately the global quality status of CR using samples collected worldwide to represent its total production. HCA and CCA used in this study provide a full scope of the correlation between growth area, macroscopic characteristics, and quality of CR, which in turn could facilitate further research into its cultivation, quality assurance, and commodity specification. More importantly, this study provides a viable research model for the comprehensive evaluation of traditional Chinese herbal medicinal materials and exploration of the correlation between their macroscopic characteristics and quality.

## Figures and Tables

**Table 1:** Original information of CR samples and morphological features (mean ± *s*, *n* = 90).

No.	Place of origins	Plant type	Geographical district	Shape	Length (cm)	Diameter (cm)	Weight (g)
S01	Hubei province	Wild	Central China	Spindle	1.6500 ± 0.4099	0.5422 ± 0.1095	0.3313 ± 0.1227
S02	Jiangxi province	Wild	Central China	Spindle	1.3233 ± 0.2295^△^	0.4167 ± 0.0922^☆^	0.1449 ± 0.0470^*∗*^
S03	Jiangxi province	Wild	Central China	Spindle	1.3922 ± 0.3905^△^	0.4656 ± 0.1056^☆^	0.2282 ± 0.1125^*∗*^
S04	Guangdong province	Cultivation	South China	Spindle	1.3544 ± 0.4251^△^	0.5089 ± 0.1082^☆^	0.3129 ± 0.1216^*∗*^
S05	Guangdong province	Cultivation	South China	Spindle	1.3933 ± 0.3224	0.4967 ± 0.0767	0.3037 ± 0.0915^*∗*^
S06	Guangdong province	Cultivation	South China	Spindle	1.6022 ± 0.3505^△^	0.4889 ± 0.0971^☆^	0.3224 ± 0.1246^*∗*^
S07	Guangdong province	Cultivation	South China	Spindle	1.8444 ± 0.3816^△^	0.5411 ± 0.0893	0.3718 ± 0.1260
S08	Guangdong province	Cultivation	South China	Spindle	2.2878 ± 0.5690^△^	0.6056 ± 0.1099^☆^	0.5609 ± 0.1813^*∗*^
S09	Shandong province	Wild	East China	Deplanate	2.1322 ± 0.5042	0.6456 ± 0.1469^☆^	0.5028 ± 0.2357
S10	Shandong province	Wild	East China	Deplanate	1.6544 ± 0.3643^△^	0.5544 ± 0.1175^☆^	0.2661 ± 0.1125^*∗*^
S11	Henan province	Wild	Central China	Spindle	1.9100 ± 0.3879^△^	0.5500 ± 0.0980^☆^	0.4047 ± 0.1437
S12	Guangxi province	Cultivation	South China	Spindle	1.7056 ± 0.2500^△^	0.5033 ± 0.0836^☆^	0.3267 ± 0.0756^*∗*^
S13	Hainan province	Cultivation	South China	Spindle	1.8500 ± 0.4510	0.5944 ± 0.0959^☆^	0.4757 ± 0.1317
S14	Anhui province	Wild	East China	Spindle	1.8256 ± 0.2904	0.5689 ± 0.0927	0.4361 ± 0.1382^*∗*^
S15	Yunnan province	Wild	Southwest China	Spindle	1.8389 ± 0.2862^△^	0.5589 ± 0.0855^☆^	0.4520 ± 0.1123^*∗*^
S16	Shanxi province	Wild	North China	Spindle	1.9633 ± 0.2811^△^	0.5922 ± 0.0763	0.5005 ± 0.1036^*∗*^
S17	Sichuan province	Wild	Southwest China	Spindle	1.8233 ± 0.3037	0.6633 ± 0.1354^☆^	0.5384 ± 0.1861
S18	Shaanxi province	Wild	Northwest China	Spindle	2.0144 ± 0.3227^△^	0.5622 ± 0.1121^☆^	0.3331 ± 0.1239^*∗*^
S19	Guangdong province	Cultivation	South China	Spindle	1.6633 ± 0.3993	0.4867 ± 0.0846	0.3061 ± 0.1302
S20	Guangxi province	Cultivation	South China	Spindle	1.8189 ± 0.3648^△^	0.5256 ± 0.0914^☆^	0.3856 ± 0.1661^*∗*^
S21	Guangxi province	Cultivation	South China	Spindle	1.8122 ± 0.4964	0.5333 ± 0.0931	0.3934 ± 0.1618^*∗*^
S22	Guangxi province	Cultivation	South China	Spindle	1.5356 ± 0.2841^△^	0.5033 ± 0.0795^☆^	0.2935 ± 0.0711^*∗*^
S23	Guangxi province	Cultivation	South China	Spindle	1.7222 ± 0.3444	0.5922 ± 0.0922	0.4596 ± 0.1260
S24	Hainan province	Cultivation	South China	Spindle	1.8311 ± 0.3504^△^	0.5811 ± 0.0905^☆^	0.4678 ± 0.1631^*∗*^
S25	Guangxi province	Cultivation	South China	Spindle	1.5467 ± 0.2930^△^	0.4944 ± 0.0821	0.2671 ± 0.0935^*∗*^
S26	Hubei province	Wild	Central China	Spindle	2.3956 ± 0.4521^△^	0.6278 ± 0.8161	0.6726 ± 0.1920^*∗*^
S27	Jiangxi province	Wild	Central China	Spindle	1.7556 ± 0.3464^△^	0.4689 ± 0.0784^☆^	0.2835 ± 0.0811^*∗*^
S28	Jiangxi province	Wild	Central China	Spindle	2.1511 ± 0.3959^△^	0.5833 ± 0.0860	0.5188 ± 0.1412^*∗*^
S29	Guangdong province	Cultivation	South China	Spindle	2.0400 ± 0.4716	0.6156 ± 0.1032	0.6913 ± 0.1962^*∗*^
S30	Guangdong province	Cultivation	South China	Spindle	2.2256 ± 0.3948^△^	0.5556 ± 0.0932^☆^	0.6243 ± 0.1395^*∗*^
S31	Guangdong province	Cultivation	South China	Spindle	1.3933 ± 0.3224^△^	0.4967 ± 0.0767^☆^	0.3037 ± 0.0915^*∗*^
S32	Guangdong province	Cultivation	South China	Spindle	2.2022 ± 0.4440^△^	0.5667 ± 0.0843	0.5818 ± 0.1044^*∗*^
S33	Guangdong province	Cultivation	South China	Spindle	3.0800 ± 0.5568	0.6600 ± 0.0987^☆^	1.0066 ± 0.3152^*∗*^
S34	Shandong province	Wild	East China	Deplanate	2.8433 ± 0.4617^△^	0.7356 ± 0.1587^☆^	1.0641 ± 0.2864^*∗*^
S35	Shandong province	Wild	East China	Deplanate	2.1856 ± 0.3626^△^	0.6444 ± 0.1107	0.6015 ± 0.1711
S36	Henan province	Wild	Central China	Spindle	2.5200 ± 0.4318^△^	0.6267 ± 0.1009	0.8139 ± 0.1919^*∗*^
S37	Guangxi province	Cultivation	South China	Spindle	2.5211 ± 0.3125^△^	0.6244 ± 0.0970^☆^	0.7565 ± 0.2022^*∗*^
S38	Hainan province	Cultivation	South China	Spindle	2.8689 ± 0.4302^△^	0.6667 ± 0.1054^☆^	0.8832 ± 0.1958^*∗*^
S39	Anhui province	Wild	East China	Spindle	2.7189 ± 0.4155^△^	0.8144 ± 0.0961^☆^	1.2104 ± 0.2493^*∗*^
S40	Yunnan province	Wild	Southwest China	Spindle	2.5667 ± 0.3276^△^	0.6056 ± 0.0993^☆^	0.7993 ± 0.2028^*∗*^
S41	Shanxi province	Wild	North China	Spindle	2.7456 ± 0.3284^△^	0.7089 ± 0.0839^☆^	0.9490 ± 0.1982^*∗*^
S42	Sichuan province	Wild	Southwest China	Spindle	2.5667 ± 0.3930	0.8167 ± 0.1276	1.2205 ± 0.2965
S43	Shaanxi province	Wild	Northwest China	Spindle	2.7400 ± 0.5444^△^	0.6578 ± 0.1801	0.6688 ± 0.2000^*∗*^
S44	Guangxi province	Cultivation	South China	Spindle	1.9422 ± 0.7619	0.5444 ± 0.0944	0.5206 ± 0.1446^*∗*^
S45	Guangxi province	Cultivation	South China	Spindle	2.5178 ± 0.4875^△^	0.6467 ± 0.1002	0.7982 ± 0.2837^*∗*^
S46	Hainan province	Cultivation	South China	Spindle	2.5133 ± 0.4124^△^	0.6778 ± 0.0986^☆^	0.8416 ± 0.2594^*∗*^
S47	Guangxi province	Cultivation	South China	Spindle	2.0678 ± 0.3768^△^	0.5522 ± 0.0792^☆^	0.4962 ± 0.1186^*∗*^

^△^means the value of length *p* < 0.05 compared to S01; ^☆^means the value of diameter *p* < 0.05 compared to S01; ^*∗*^means the value of weight *p* < 0.05 compared to S01.

**Table 2:** Specific batch of CR in different categories of HCA.

Categories	Figure 7(a)	Figure 7(b)	Figure 7(c)	Figure 7(d)
Category 1	S01, S02, S03, S05, S07, S09, S12, S14, S15, S17, S18, S19, S20, S21, S22, S26, S27, S28, S29, S30, S31, S32, S35, S36, S37, S40, S42, S43, S44	S01, S02, S04, S05, S06, S07, S09, S11, S12, S15, S17, S18, S19, S21, S22, S25, S27, S28, S29, S30, S31, S32, S33, S42, S40, S44	S04, S05, S07, S12, S29, S20, S21, S22, S29, S30, S32, S37	S04, S05, S06, S07, S12, S19, S21, S22, S25, S29, S30, S31, S32, S33, S44
Category 2	S04, S10, S11, S34	S10, S20, S26, S34, S35, S36, S37, S39, S43	S06, S31	S20, S37
Category 3	S06	S03	S08, S13, S23, S24, S25, S33, S38, S44, S45, S46, S47	S08, S13, S23, S24, S38, S45, S46, S47
Category 4	S08, S13, S16, S23, S24, S25, S33, S38, S39, S41, S45, S46, S47	S08, S13, S14, S16, S23, S24, S38, S41, S45, S46, S47	—	—

**Table 3:** Cross load analysis of variables in vector 1.

Variable	1	2	3
Length	0.474	0.224	−0.128
Diameter	0.523	0.282	0.008
Weight	0.346	0.328	−0.061

**Table 4:** Cross load analysis of variables in vector 2.

Variable	1	2	3
Essential oil	−0.122	0.080	−0.070
Cyperotundone	−0.014	0.149	−0.210
*α*−Cyperone	−0.127	−0.288	0.151
Nootkatone	−0.450	−0.134	0.015
Ethanol extracts	0.604	−0.115	−0.095

## Data Availability

The data used to support the findings of this study are included within the article.

## Abbreviations

BHC: Hexachlorocyclohexane
CCA: Canonical correlation analysis
ChP: Chinese Pharmacopeia
CR: Cyperi Rhizoma
DDT: Dichlorodiphenyltrichloroethane
HCA: Hierarchical cluster analysis
HPLC: High-performance liquid chromatography
TCM: Traditional Chinese medicine
TLC: Thin-layer chromatography.

## References

[1] EBOCFOCAO Sciences (1961). *Chinese Flora*.

[2] Tajadini H., Saifadini R., Choopani R., Mehrabani M., Kamalinejad M., Haghdoost A. A. (2015). Herbal medicine Davaie Loban in mild to moderate Alzheimer’s disease: a 12-week randomized double-blind placebo-controlled clinical trial. *Complementary Therapies in Medicine*.

[3] Choi J. E., Park D. M., Chun E. (2017). Control of stress-induced depressive disorders by So-ochim-tang-gamibang, a Korean herbal medicine. *Journal of Ethnopharmacology*.

[4] Salunke M., Deshpande M., Bhalerao S. (2017). Experiential documentation of Trimad for its anti-obesity potential: a survey of ayurvedic physicians from Pune city. *Journal of Ayurveda and Integrative Medicine*.

[5] Sahib A. S. (2013). Treatment of irritable bowel syndrome using a selected herbal combination of Iraqi folk medicines. *Journal of Ethnopharmacology*.

[6] Kilani S., Ammar R. B., Bouhlel I. (2005). Investigation of extracts from (Tunisian) Cyperus rotundus as antimutagens and radical scavengers. *Environmental Toxicology and Pharmacology*.

[7] Mengi S. A., Patel P. P. (2008). Assessment of hydroalcoholic extract of *Cyperus rotundus* in high fat diet induced hyperlipidaemia in rats. *Atherosclerosis Supplements*.

[8] Yagi S., Babiker R., Tzanova T., Schohn H. (2016). Chemical composition, antiproliferative, antioxidant and antibacterial activities of essential oils from aromatic plants growing in Sudan. *Asian Pacific Journal of Tropical Medicine*.

[9] NP Commission (2015). *Chinese Pharmacopoeia*.

[10] Hemanth Kumar K., Razack S., Nallamuthu I., Khanum F. (2014). Phytochemical analysis and biological properties of *Cyperus rotundus* L. *Industrial Crops and Products*.

[11] Pirzada A. M., Ali H. H., Naeem M., Latif M., Bukhari A. H., Tanveer A. (2015). Cyperus rotundus L.: Traditional uses, phytochemistry, and pharmacological activities. *Journal of Ethnopharmacology*.

[12] Kilani S., Ben Sghaier M., Limem I. (2008). In vitro evaluation of antibacterial, antioxidant, cytotoxic and apoptotic activities of the tubers infusion and extracts of Cyperus rotundus. *Bioresource Technology*.

[13] Seo E. J., Lee D.-U., Kwak J. H., Lee S.-M., Kim Y. S., Jung Y.-S. (2011). Antiplatelet effects of Cyperus rotundus and its component (+)-nootkatone. *Journal of Ethnopharmacology*.

[14] Azimi A., Ghaffari S. M., Riazi G. H., Arab S. S., Tavakol M. M., Pooyan S. (2016). *α*-Cyperone of Cyperus rotundus is an effective candidate for reduction of inflammation by destabilization of microtubule fibers in brain. *Journal of Ethnopharmacology*.

[15] Rabiei Z., Hojjati M., Rafieian-Kopaeia M., Alibabaei Z. (2013). Effect of Cyperus rotundus tubers ethanolic extract on learning and memory in animal model of Alzheimer. *Biomedicine and Aging Pathology*.

[16] Ngamrojanavanich N., Manakit S., Pornpakakul S., Petsom A. (2006). Inhibitory effects of selected Thai medicinal plants on Na^+^,K^+^-ATPase. *Fitoterapia*.

[17] Hemanth Kumar K., Tamatam A., Pal A., Khanum F. (2013). Neuroprotective effects of Cyperus rotundus on SIN-1 induced nitric oxide generation and protein nitration: ameliorative effect against apoptosis mediated neuronal cell damage. *Neurotoxicology*.

[18] Kumar K. H., Khanum F. (2013). Hydroalcoholic extract of Cyperus rotundus Ameliorates H2O2-induced Human neuronal cell damage via its anti-oxidative and anti-apoptotic machinery. *Cellular and Molecular Neurobiology*.

[19] Zhang L. L., Zhang L. F., Hu Q. P., Hao D. L., Xu J. G. (2017). Chemical composition, antibacterial activity of Cyperus rotundus rhizomes essential oil against *Staphylococcus aureus* via membrane disruption and apoptosis pathway. *Food Control*.

[20] Mohamed G. A. (2015). Iridoids and other constituents from Cyperus rotundus L. rhizomes. *Bulletin of Faculty of Pharmacy Cairo University*.

[21] Badgujar S. B., Bandivdekar A. H. (2015). Evaluation of a lactogenic activity of an aqueous extract of Cyperus rotundus Linn. *Journal of Ethnopharmacology*.

[22] Boyd N. S., Vallad G., Wu F., Noling J., Guan Z. (2017). Placement of metam potassium in combination with dimethyl disulfide, chloropicrin, and 1,3-dichloropropene for Cyperus rotundus L. and broadleaf weed control in tomato (Solanum lycopersicum L.). *Crop Protection*.

[23] Gilreath J. P., Jones J. P., Santos B. M., Overman A. J. (2013). Soil fumigant evaluations for soilborne pest and *Cyperus rotundus* control in fresh market tomato. *Crop Protection*.

[24] Kadir J., Charudattan R. (2000). Dactylaria higginsii, a Fungal Bioherbicide agent for purple Nutsedge (*Cyperus rotundus*). *Biological Control*.

[25] Gilreath J. P., Santos B. M. (2004). Herbicide dose and incorporation depth in combination with 1,3-dichloropropene plus chloropicrin for *Cyperus rotundus* control in tomato and pepper. *Crop Protection*.

[26] Hussain I., Singh N. B., Singh A., Singh H. (2016). Allelopathic potential of sesame plant leachate against Cyperus rotundus L. *Annals of Agrarian Science*.

[27] Lu J. R., Li W. B., Fu C. M. (2015). Investigation on quality control of medicinal material of Cyperi Rhizoma. *Journal of Chinese hospital pharmacy*.

[28] Jiang L. J., Pi S. L., Fang T. Z., Yao J. X., Xu Z. D., Lin L. M. (2018). Study on the HPLC fingerprint of Cyperi rhizoma. *Chinese Modern Medicine*.

[29] Wang S. Y., Li W. B., Lu J. R. (2015). Determination of cyperotundone, nootkatone, and *α*-cyperone in Cyperus rotundus L. from different growing areas by HPLC. *Chinese traditional patent medicine*.

[30] Tsoyi K., Jang H. J., Lee Y. S. (2011). (+)-Nootkatone and (+)-valencene from rhizomes of Cyperus rotundus increase survival rates in septic mice due to heme oxygenase-1 induction. *Journal of Ethnopharmacology*.

[31] Wang Q., Liang Z., Peng Y. (2015). Whole transverse section and specific-tissue analysis of secondary metabolites in seven different grades of root of Paeonia lactiflora using laser microdissection and liquid chromatography-quadrupole/time of flight-mass spectrometry. *Journal of Pharmaceutical and Biomedical Analysis*.

[32] Chen Y. J., Liang Z. T., Zhu Y. (2014). Tissue-specific metabolites profiling and quantitative analyses of flavonoids in the rhizome of Belamcanda chinensis by combining laser-microdissection with UHPLC-Q/TOF-MS and UHPLC-QqQ-MS. *Talanta*.

[33] Yi L., Liang Z. T., Peng Y., Yao X., Chen H. B., Zhao Z. Z. (2012). Tissue-specific metabolite profiling of alkaloids in Sinomenii Caulis using laser microdissection and liquid chromatography-quadrupole/time of flight-mass spectrometry. *Journal of Chromatography A*.

[34] Chen Q. L., Chen Y. J., Zhou S. S. (2018). Laser microdissection hyphenated with high performance gel permeation chromatography-charged aerosol detector and ultra performance liquid chromatography-triple quadrupole mass spectrometry for histochemical analysis of polysaccharides in herbal medicine: Ginseng, a case study. *International Journal of Biological Macromolecules*.

